# Optimal body size adjustment of L3 CT skeletal muscle area for sarcopenia assessment

**DOI:** 10.1038/s41598-020-79471-z

**Published:** 2021-01-11

**Authors:** Brian A. Derstine, Sven A. Holcombe, Brian E. Ross, Nicholas C. Wang, Grace L. Su, Stewart C. Wang

**Affiliations:** Michigan Medicine, Ann Arbor, MI USA

**Keywords:** Ageing, Skeletal muscle, Risk factors, Diagnostic markers

## Abstract

Measurements of skeletal muscle cross-sectional area (SMA) at the level of the third lumbar (L3) vertebra derived from clinical computed tomography (CT) scans are commonly used in assessments of sarcopenia, the loss of skeletal muscle mass and function associated with aging. As SMA is correlated with height and Body Mass Index (BMI), body size adjustment is necessary to fairly assess sarcopenic low muscle mass in individuals of different height and BMI. The skeletal muscle index, a widely used measure, adjusts for height as $(SMA/height^2)$ but uses no BMI adjustment. There is no agreed upon standard for body size adjustment. We extracted L3 SMA using non-contrast-enhanced CT scans from healthy adults, split into ‘Under-40’ and ‘Over-40’ cohorts. Sex-specific allometric analysis showed that height to the power of one was the optimal integer coefficient for height adjusted SMA in both males and females. We computed two height-adjusted measures $SMA_{HT}=SMA/height$ and $SMA_{HT2}=SMA/height^2$, comparing their Pearson correlations versus age, height, weight, and BMI separately by sex and cohort. Finally, in the ‘Under-40’ cohort, we used linear regression to convert each height-adjusted measure into a z-score ($z(SMA_{HT})$, $z(SMA_{HT2})$) adjusted for BMI. $SMA_{HT}$ was less correlated with height in both males and females ($r=0.005$, $p=0.91$ and $r=0.1$, $p=0.01$) than $SMA_{HT2}$ ($r=-\,0.30$ and $r=-\,0.21$, $p<0.001$). $z(SMA_{HT})$ was uncorrelated with BMI and weight, and minimally correlated with height in males and females ($r=-\,0.01$, $p=0.85$ and $r=0.15$, $p<0.001$). The final $z(SMA_{HT})$ equation was: $z = (I - {\widehat{I}}) / SD(I)$, where $I = SMA/height$, ${\widehat{I}} = 50 + BMI + 13 \times sex + 0.6 \times BMI \times sex$, $SD(I) = 8.8 + 2.6 \times sex$, and *sex* = 1 if male, 0 if female. We propose $SMA_{HT}$ for optimal height adjustment and the $z(SMA_{HT})$ score for optimal height and BMI adjustment. By minimizing correlations with height and BMI, the $z(SMA_{HT})$ score produces unbiased assessments of relative L3 skeletal muscle area across the full range of body sizes.

## Introduction

The European Working Group on Sarcopenia in Older People (EWGSOP) defines sarcopenia as the loss of both muscle mass and function^1^. This manuscript directly addresses only the muscle mass half of that definition. Computed tomography (CT) measurements of skeletal muscle cross-sectional area (SMA) performed at the third lumbar vertebra (L3) are prevalent in assessments of sarcopenic low muscle mass^2–12^, though other vertebrae or muscle groups have been used^13–16^. Cutoffs for sarcopenic muscle quantity set at two standard deviations below the mean of a healthy, young adult population were recommended by EWGSOP. The 18 (or 20) to 40 age range has been widely used to define ‘young, adult’ reference populations^12,15–21^, and is further supported by the observation that muscle mass loss increases after age 40. Revised EWGSOP guidelines note that ‘fundamentally, muscle mass is correlated with body size; i.e., individuals with a larger body size normally have larger muscle mass’^22^, and identify three examples of body size adjustment: dividing muscle mass by height-squared, by weight, or by BMI directly with muscle measurements derived from CT, dual-energy X-ray absorptiometry (DXA), or bioelectrical impedance analysis (BIA)^18,23^. However, no specific recommendation for body size adjustment was made^22^.

The purpose of body size adjustment is to remove the association between the original measure and a biologically related body size measure, thus enabling unbiased comparisons to be made across the range of body sizes. For example, human body mass (*weight*) scales approximately with $height^2$ so a relative weight index, Body Mass Index (BMI), was created and validated as $BMI = weight/height^2$ using allometric analysis^24–26^. By design, BMI is uncorrelated with height and as such, enables unbiased comparisons of body mass between individuals of different heights. Similar to weight, L3 SMA is correlated with height; so a relative (for height) skeletal muscle index ($SMI=SMA/height^2$) was developed based upon the work of Baumgartner et al. with DXA measurements^2,4,9,15,27,28^. However, the height adjustment used in L3 SMI may be neither optimal nor sufficient. Our analysis shows that CT-derived L3 SMI remains correlated with height, suggesting that height-squared is not the optimal height adjustment factor. Furthermore, multiple studies have noted that SMI is correlated with BMI^29,30^, suggesting that height adjustment is insufficient and that BMI adjustment is also warranted. Diagnosis of sarcopenic low muscle mass using fixed cutoff values that have been adjusted for height but not BMI can result in under-diagnosis in high BMI individuals with relatively low muscle (i.e., sarcopenic obese) and over-diagnosis in low BMI individuals with relatively normal muscle quantity (i.e., healthy lean)^9^. Despite this fact, an appropriately BMI-adjusted CT skeletal muscle index is not widely used.

Attempts to adjust sarcopenia cutoffs to address the association between SMI and BMI include: Newman et al. proposed a method based on the residuals of a linear regression model in an elderly cohort^29^; Martin et al. proposed different cutoffs for high BMI ($>25$) males, but not females in an elderly cancer cohort^5^; Zhuang et al. proposed Asian (Wenzhou, China) cutoffs for males and females with a low mean BMI^9^; and Van der Werf et al. provided sex-, age-, and BMI-specific predicted percentiles for SMA and SMI in healthy subjects between age 20–60 and 20–82^11^.

Sarcopenia is a condition that may exist at any body mass, and properly adjusting muscle quantity measures for body mass is widely recognized as necessary^22,31,32^. Human body mass (weight) is determined by height and absolute skeletal muscle quantity, along with fat, bone, and other tissue. Since height and weight are correlated, height and BMI (which are uncorrelated) should be the primary targets for body size adjustment. Therefore, we suggest that the optimal body size adjusted skeletal muscle index meet two simple criteria: it should be uncorrelated with (1) height and (2) BMI in a young, healthy reference population. By doing so, the index excludes the variation in muscle quantity explained by height and BMI (relative weight). The resulting relative muscle index distinguishes between ‘more muscular’ and ‘less muscular’ body compositions at any BMI. We suggest that this index would optimally quantify sarcopenic low muscle mass across the full range of human body sizes; it would be unbiased in tall, short, thin, or obese individuals.

We previously published fixed sarcopenia cutoffs for SMA and SMI, however, we did not assess whether dividing SMA by $height^2$ resulted in a measure that was uncorrelated with height, nor did we address the underlying association between SMI and BMI^12,15^. In this manuscript, we focus on CT measurements of skeletal muscle cross-sectional area at the third lumbar vertebra (L3). Our aim is to determine the optimal body size adjusted L3 skeletal muscle index that meets the above criteria, i.e, it is uncorrelated with height and BMI in a young, healthy population.

## Results

### Population summary

On average, the ‘Under-40’ cohort males and females had greater muscle quantity than those in the ‘Over-40’ cohort ($p<0.001$), were slightly taller ($p<0.07$), but were not significantly heavier by weight or BMI ($p>0.23$) (Table 1). Females were 1.64 meters tall with a BMI of 27 on average, while males were 1.79 meters with a BMI of 28 in both cohorts. Mean SMA was 7.7% lower in females, and 4.5% lower in males in the ‘Over-40’ versus ‘Under-40’ cohort. The average z-scores were significantly lower in the ‘Over-40’ cohort compared to the ‘Under-40’ cohort, reflecting increased sarcopenia in the older group.

**Table 1:** Sex- and cohort-specific demographics and skeletal muscle measures.

Sex	Variable	Under-40			Over-40			*p*
		N	Mean	s.d.	N	Mean	s.d.	
F	Age (year)	610	31.06	5.90	512	49.73	6.36	<**0.001**
	Height (m)	610	1.64	0.07	512	1.64	0.06	0.020
	Weight (kg)	610	73.68	15.79	512	72.66	12.97	0.237
	BMI (kg/m^2^)	610	27.22	5.41	512	27.17	4.58	0.863
	Underweight	8	1.3%		0	0%		
	Normal	241	39.5%		181	35.4%		
	Overweight	167	27.4%		196	38.3%		
	Obese class I	138	22.6%		104	20.3%		
	Obese class II	45	7.4%		28	5.5%		
	Obese class III	11	1.8%		3	0.6%		
	Race	610			512			**0.005**
	African American	65	10.7%		33	6.4%		
	Asian	8	1.3%		4	0.8%		
	Caucasian	339	55.6%		291	56.8%		
	Other	22	3.6%		7	1.4%		
	Unknown	176	28.9%		177	34.6%		
	*SMA* (cm^2^/)	610	130.09	18.78	512	120.07	15.84	<**0.001**
	*SMA*/*ht* (cm^2^/m)	610	79.08	10.55	512	73.43	9.26	<**0.001**
	$SMA/ht^2$ (cm^2^/m^2^)	610	48.15	6.52	512	44.97	5.94	<**0.001**
	*z*(*SMA*/*ht*)	610	0.00	1.00	512	− 0.63	0.94	<**0.001**
	$z(SMA/ht^2)$	610	0.00	1.00	512	− 0.58	0.98	<**0.001**
M	Age (year)	448	30.29	5.81	279	49.64	6.73	<**0.001**
	Height (m)	448	1.79	0.07	279	1.78	0.07	0.071
	Weight (kg)	448	88.87	16.85	279	88.00	12.72	0.427
	BMI (kg/m^2^)	448	27.70	4.67	279	27.73	3.37	0.918
	Underweight	2	0.4%		0	0%		
	Normal	126	28.1%		61	21.9%		
	Overweight	198	44.2%		154	55.2%		
	Obese class I	83	18.5%		57	20.4%		
	Obese class II	36	8.0%		7	2.5%		
	Obese class III	3	0.7%		0	0%		
	Race	448			279			**0.008**
	African American	46	10.3%		12	4.3%		
	Asian	7	1.6%		1	0.4%		
	Caucasian	244	54.5%		147	52.7%		
	Other	10	2.2%		9	3.2%		
	Unknown	141	31.5%		110	39.4%		
	*SMA* (cm^2^/)	448	195.61	25.91	279	186.74	24.08	<**0.001**
	*SMA*/*ht* (cm^2^/m)	448	109.31	13.79	279	104.89	12.72	<**0.001**
	$SMA/ht^2$ (cm^2^/m^2^)	448	61.18	8.12	279	59.01	7.44	<**0.001**
	*z*(*SMA*/*ht*)	448	0.00	1.00	279	− 0.39	0.95	<**0.001**
	$z(SMA/ht^2)$	448	0.00	1.00	279	− 0.32	0.93	<**0.001**

p-values less than 0.01 shown in bold.

### Body size adjustment

Allometric analysis of weight versus height resulted in optimal coefficients of 2.07 (male) and 1.84 (female), or 2 when rounded to the nearest integer. SMA versus height analysis resulted in optimal coefficients of 1.02 (males) and 1.33 (females), or 1 when rounded to the nearest integer.

**Figure 1:**
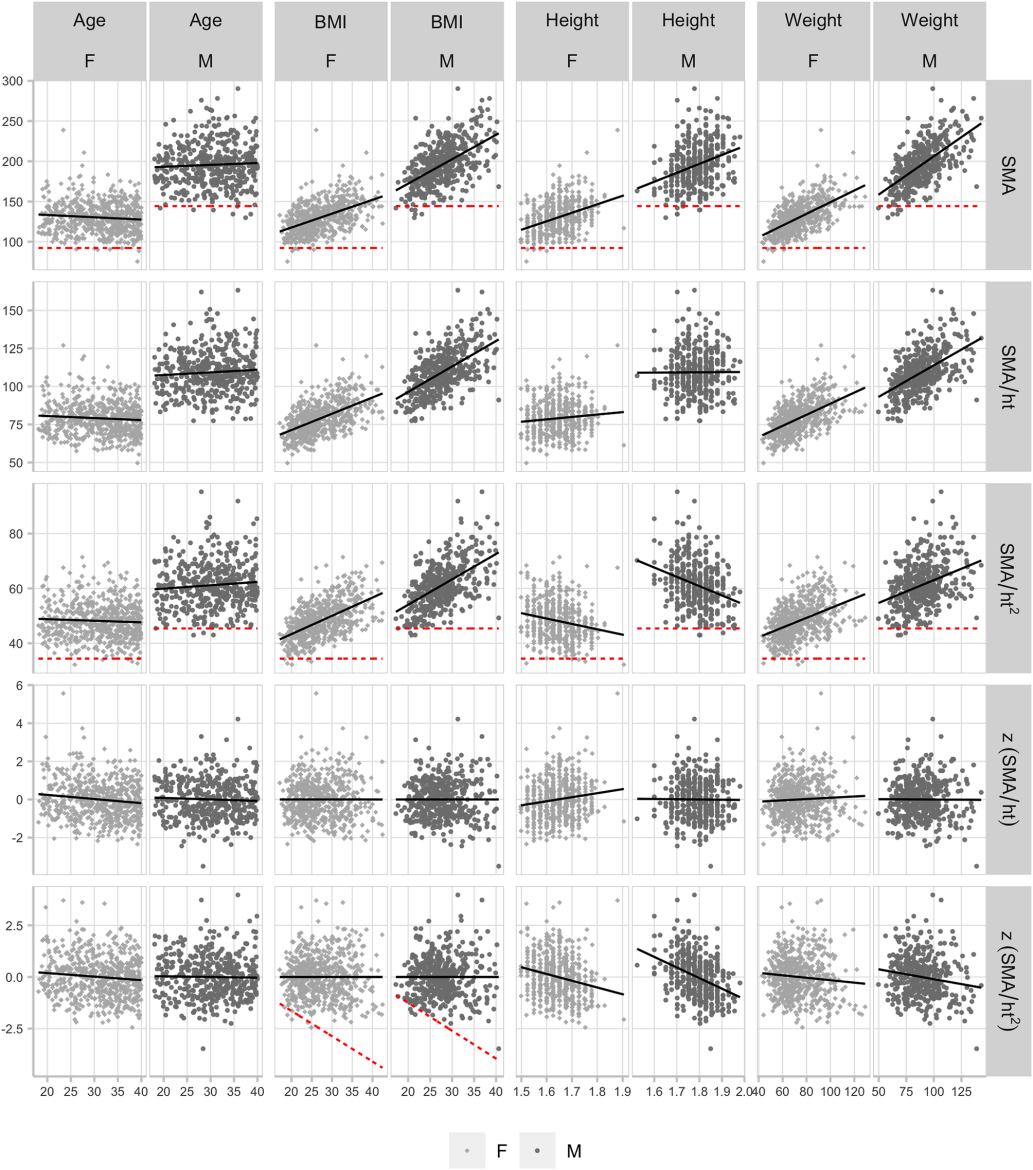
Under-40 cohort: scatter plots of L3 skeletal muscle area measures versus age, BMI, height, and weight, split by sex. Best-fit linear regression line overlaid (black, solid), and previously reported (Derstine, 2018)^15^ sarcopenia ‘fixed’ cutoff values (red, dashed) where applicable.

**Figure 2:**
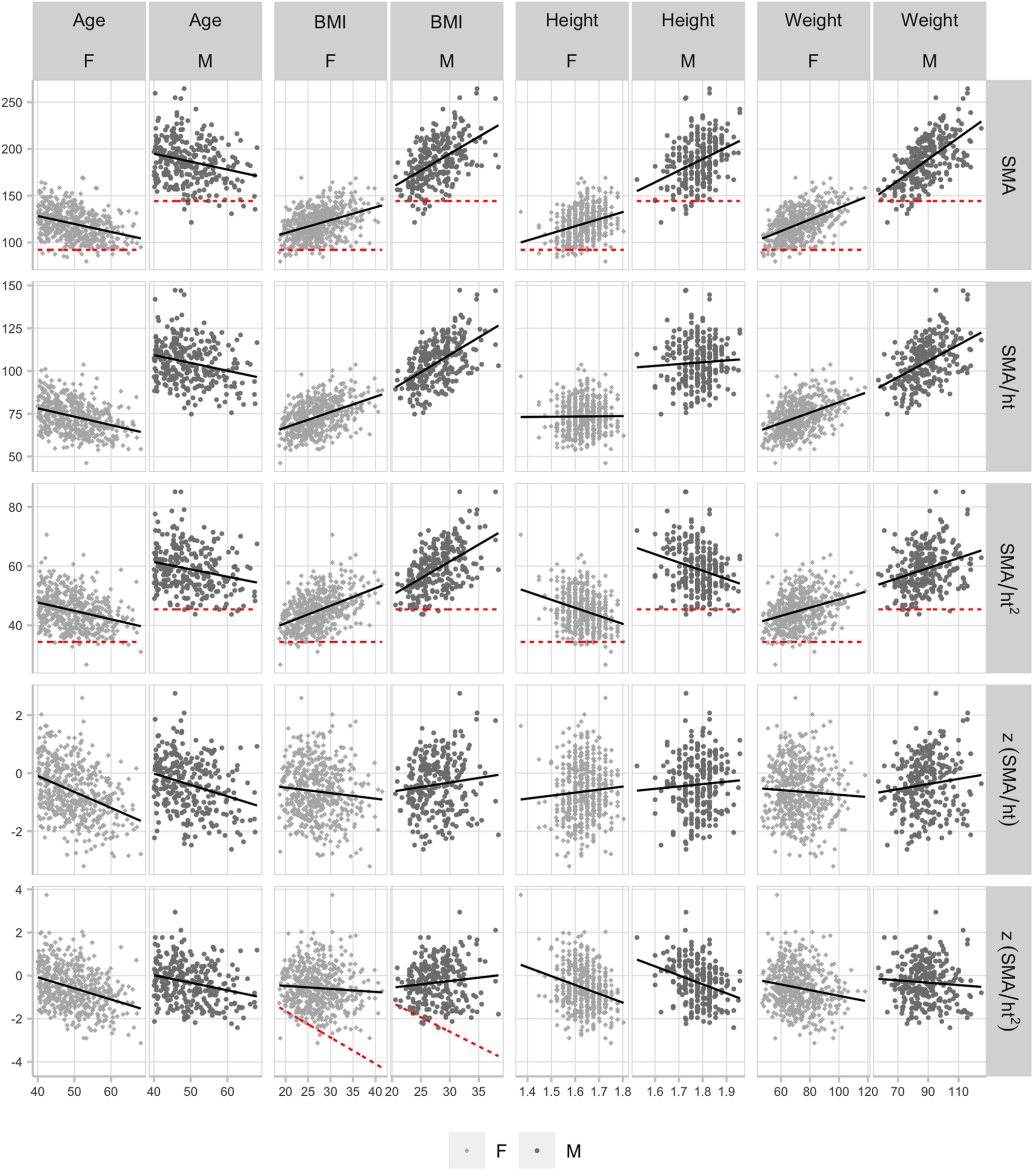
Over-40 cohort: Scatter plots of L3 skeletal muscle area measures versus age, BMI, height, and weight, split by sex. Best-fit linear regression line overlaid (black, solid), and Derstine, 2018^15^ sarcopenia ‘fixed’ cutoff values (red, dashed) shown for SMA, $SMA_{HT2}$, and $z(SMA_{HT2})$ vs. BMI.

Unadjusted L3 SMA and height-adjusted SMA measures were differently correlated with age, BMI, height, and weight in both cohorts (Figs. 1, 2). In the ‘Under-40’ cohort, SMA, $SMA_{HT2}$, and $SMA_{HT}$ were uncorrelated with age (Table 2). SMA, $SMA_{HT2}$, and $SMA_{HT}$ were positively correlated with BMI and weight. SMA was positively correlated with height and $SMA_{HT2}$ was negatively correlated with height. $SMA_{HT}$ correlations with height were closer to zero than $SMA_{HT2}$. Of the z-scores, $z(SMA_{HT2})$ was uncorrelated with age and BMI, and negatively correlated with height and weight, though the age ($r=-\,0.101$, $p<0.012$) and weight ($r=-\,0.094$, $p<0.021$) correlations were borderline in females. $z(SMA_{HT})$ was uncorrelated with age, BMI, height, and weight in males, and in females was uncorrelated with BMI and weight, negatively correlated with age, and positively correlated with height. The $z(SMA_{HT})$ correlation with height was closer to zero than $z(SMA_{HT2})$.

In the ‘Over-40’ cohort, all measures were more strongly and negatively correlated with age (Table 2). As in the younger cohort, SMA, $SMA_{HT2}$, and $SMA_{HT}$ were positively correlated with BMI and weight. SMA was positively correlated with height and $SMA_{HT2}$ was negatively correlated with height. $SMA_{HT}$ was uncorrelated with height. $z(SMA_{HT2})$ was uncorrelated with BMI, and negatively correlated with height and weight, though the correlation did not reach significance in males ($r=-\,0.075$, $p=0.212$). $z(SMA_{HT})$ in the older cohort was uncorrelated with height, weight, and BMI.

In supplemental analysis, we explored direct adjustment for BMI and weight without the use of regression. For the weight and BMI alternative measurements, *SMA*/*BMI* and *SMA*/*wt* were significantly lower in the ‘Over-40’ versus ‘Under-40’ cohort (Table S2), and both were negatively correlated with age, BMI, and weight in both cohorts (Table S2, Fig. S1). *SMA*/*wt* was negatively correlated with height, and *SMA*/*BMI* was positively correlated with height (Table S2). These results suggest that dividing SMA by BMI or weight directly would not be optimal.

**Table 2:** Sex-specific Pearson correlation, p-value, and 95% CI shown for each L3 skeletal muscle area measure versus age, BMI, height, and weight for each cohort.

Cohort	Variable	Sex	N	Age	BMI	Height	Weight
Under-40	*SMA*	F	610	− 0.089 p=0.028	**0.495 p**<**0.001**	**0.383 p**<**.001**	**0.610 p**<**0.001**
				(− 0.167, − 0.009)	**(0.433, 0.553)**	**(0.314, 0.449)**	**(0.558, 0.658)**
		M	448	0.053 p=0.259	**0.547 p**<**.001**	**0.308 p**<**0.001**	**0.619 p**<**0.001**
				(− 0.039, 0.145)	**(0.478, 0.608)**	**(0.222, 0.390)**	**(0.558, 0.673)**
	*SMA*/*ht*	F	610	− 0.075 p=0.065	**0.548 p**<**0.001**	0.102 p=0.012	**0.546 p**<**0.001**
				(− 0.153, 0.005)	**(0.490, 0.601)**	(0.023, 0.180)	**(0.488, 0.600)**
		M	448	0.073 p=0.121	**0.567 p**<**0.001**	0.005 p=0.912	**0.506 p**<**0.001**
				(− 0.019, 0.165)	**(0.500, 0.626)**	(− 0.087, 0.098)	**(0.434, 0.572)**
	$SMA/ht^2$	F	610	− 0.052 p=0.200	**0.552 p**<**0.001**	**− 0.205 p**<**0.001**	**0.428 p**<**0.001**
				(− 0.131, 0.028)	**(0.494, 0.605)**	**(− 0.280, − 0.128)**	**(0.360, 0.490)**
		M	448	0.087 p=0.064	**0.532 p**<**.001**	**− 0.301 p**<**0.001**	**0.344 p**<**0.001**
				(− 0.005, 0.179)	**(0.463, 0.596)**	**(− 0.383, − 0.214)**	**(0.260, 0.423)**
	*z*(*SMA*/*ht*)	F	610	**− 0.128 p=0.002**	0.000 p=1.000	**0.145 p**<**0.001**	0.053 p=0.189
				**(− 0.205, − 0.049)**	(− 0.079, 0.079)	**(0.066, 0.222)**	(− 0.026, 0.132)
		M	448	− 0.045 p=0.347	0.000 p=1.000	− 0.009 p=0.848	− 0.006 p=0.899
				(− 0.137, 0.048)	(− 0.093, 0.093)	(− 0.102, 0.084)	(− 0.099, 0.087)
	$z(SMA/ht^2)$	F	610	− 0.101 p=0.012	0.000 p=1.000	**− 0.223 p**<**0.001**	− 0.094 p=0.021
				(− 0.179, − 0.022)	(− 0.079, 0.079)	**(− 0.297, − 0.146)**	(− 0.172, − 0.014)
		M	448	− 0.019 p=0.691	0.000 p=1.000	**− 0.369 p**<**0.001**	**− 0.161 p=0.001**
				(− 0.111, 0.074)	(− 0.093, 0.093)	**(− 0.447, − 0.287)**	**(− 0.250, − 0.069)**
Over-40	*SMA*	F	512	**− 0.341 p**<**0.001**	**0.397 p**<**0.001**	**0.300 p**<**0.001**	**0.508 p**<**0.001**
				**(− 0.415, − 0.262)**	**(0.321, 0.467)**	**(0.219, 0.377)**	**(0.440, 0.569)**
		M	279	**− 0.237 p**<**0.001**	**0.506 p**<**0.001**	**0.356 p**<**0.001**	**0.608 p**<**0.001**
				**(− 0.345, − 0.123)**	**(0.413, 0.588)**	**(0.249, 0.454)**	**(0.529, 0.677)**
	*SMA*/*ht*	F	512	**− 0.339 p**<**0.001**	**0.445 p**<**0.001**	0.009 p=0.847	**0.426 p**<**0.001**
				**(− 0.413, − 0.259)**	**(0.373, 0.512)**	(− 0.078, 0.095)	**(0.352, 0.494)**
		M	279	**− 0.242 p**<**0.001**	**0.538 p**<**0.001**	0.057 p=0.339	**0.475 p**<**0.001**
				**(− 0.349, − 0.128)**	**(0.449, 0.616)**	(− 0.060, 0.174)	**(0.379, 0.561)**
	$SMA/ht^2$	F	512	**− 0.305 p**<**0.001**	**0.455 p**<**0.001**	**− 0.286 p**<**0.001**	**0.305 p**<**0.001**
				**(− 0.382, − 0.225)**	**(0.383, 0.521)**	**(− 0.364, − 0.205)**	**(0.225, 0.382)**
		M	279	**− 0.224 p**<**0.001**	**0.516 p**<**0.001**	**− 0.257 p**<**0.001**	**0.290 p**<**0.001**
				**(− 0.333, − 0.109)**	**(0.425, 0.597)**	**(− 0.364, − 0.144)**	**(0.179, 0.394)**
	*z*(*SMA*/*ht*)	F	512	**− 0.371 p**<**0.001**	− 0.093 p=0.035	0.069 p=0.119	− 0.055 p=0.210
				**(− 0.443, − 0.294)**	(− 0.178, − 0.007)	(− 0.018, 0.155)	(− 0.141, 0.031)
		M	279	**− 0.277 p**<**0.001**	0.111 p=0.064	0.059 p=0.323	0.119 p=0.048
				**(− 0.382, − 0.165)**	(− 0.006, 0.226)	(− 0.058, 0.176)	(0.001, 0.233)
	$z (SMA/ht^2)$	F	512	**− 0.337 p**<**0.001**	− 0.065 p=0.139	**− 0.263 p**<**0.001**	**− 0.175 p**<**0.001**
				**(− 0.412, − 0.258)**	(− 0.151, 0.021)	**(− 0.342, − 0.180)**	**(− 0.258, − 0.090)**
		M	279	**− 0.253 p**<**.001**	0.112 p=0.061	**− 0.306 p**<**0.001**	− 0.075 p=0.212
				**(− 0.359, − 0.139)**	(− 0.005, 0.227)	**(− 0.409, − 0.196)**	(− 0.191, 0.043)

p-value less than 0.01 shown in bold.

## Discussion

We analyzed two height-adjusted skeletal muscle area measures ($SMA_{HT2} = SMA/height^2$ and $SMA_{HT}=SMA/height$) and converted these into BMI-adjusted z-scores, assessing the correlation between each of these measures and age, height, weight, and BMI.

Allometric analysis confirmed that weight scales with height to the integer power of two, though the height coefficient for females (1.84) was further from the integer than males (2.07). Additionally, we found that L3 SMA scales with height to the integer power of one, and not two as has been commonly used. Once again, the coefficient for females (1.33) was further from the integer than males (1.02). These deviations explain why the height adjustment in $SMA_{HT}$ does not fully remove the correlation with height in females.

**Figure 3:**
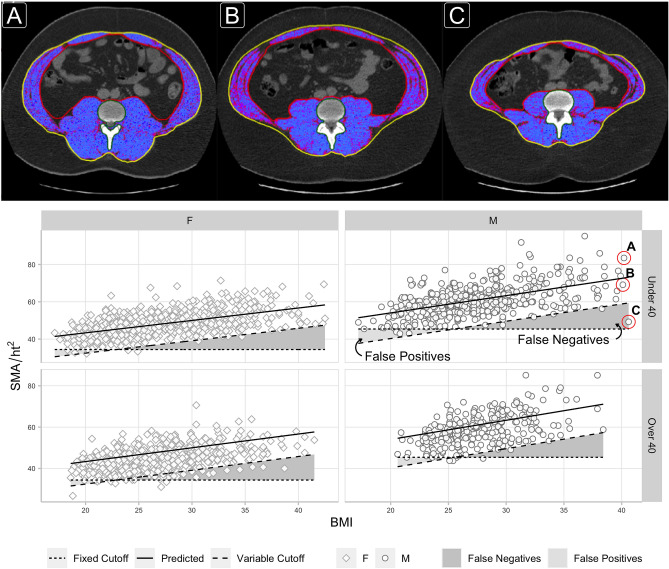
L3 axial CT images highlighting skeletal muscle area for three males of similar age (28–29) and BMI 40.1–40.6, but different $z(SMA_{HT2})$ scores (A = 1.56, B = − 0.51, C = − 3.47) are compared. The third individual (C) has a muscle area more than 3 s.d. below the mean value expected for a BMI of 40. He would be classified as not sarcopenic using the fixed cutoff, but would be sarcopenic using the variable (z-score) cutoff. Scatter plots show L3 skeletal muscle area divided by height-squared ($SMA_{HT2}$) versus BMI, split by sex and cohort. Lines overlaid for (1) the mean value predicted by our linear regression model (solid line), (2) previously reported (Derstine, 2018)^15^ sarcopenia ‘fixed cutoff’ values (dotted line), and (3) bmi-adjusted ‘variable cutoff’ values (dashed line) computed as two s.d. below the predicted mean. Regions of ‘False Positives’ and ‘False Negatives’ sarcopenia diagnosis are shown based on the difference between the ‘fixed’ and ‘variable’ cutoffs.

Following current practice, using a power of two to normalize SMA for height produces a measure ($SMA_{HT2}$) that retains a significant negative correlation with height in both males ($r=-\,0.301$) and females ($r=-\,0.205$), resulting in cutoff values that are biased towards identifying sarcopenia in taller individuals (Fig. 1). This measure also retains a significant positive correlation with BMI in both males ($r=0.532$) and females ($r=0.552$), and because cutoff values are constant for all BMI values, the cutoffs are additionally biased to identify sarcopenia in those with low BMI (Fig. 3). Specifically, these ‘fixed’ cutoffs appear prone to falsely identifying sarcopenia in low BMI individuals with muscle mass between − 1 to − 2SD of what would be expected relative to their BMI (false positives), while failing to identify sarcopenia in high BMI individuals with muscle mass more than − 2SD below what would be expected relative to their BMI (false negatives) (Fig. 3). A similar argument could be made against using ‘fixed’ cutoffs computed from $SMA_{HT}$ (Fig. S2). Conversely, ‘variable’ cutoffs based on the z-score increase linearly with BMI, following the natural distribution of muscle mass with BMI, and better identify low muscle mass at any height and BMI.

Based on our analysis, we suggest using $SMA_{HT}$ and its z-score in sarcopenia analyses for several reasons. First, using a power of one to normalize SMA for height ($SMA_{HT}$) results in a measure that is minimally correlated with height. Second, subtracting the regression model’s predicted mean value and dividing by the standard deviation produces the $z(SMA_{HT})$ score that is uncorrelated with BMI (by design). Third, the $z(SMA_{HT})$ score is negatively correlated with age in the ‘Over-40’ cohort, which is desirable, as it ensures that sarcopenia prevalence increases with age, as would be expected. Fourth, since z-scores have mean 0 and standard deviation 1, they are quite easy to interpret—values above zero indicate ‘more muscular’ BMI and values below zero indicate ‘less muscular’ BMI. Furthermore, they can be directly compared since they use common units of standard deviations. Finally, the $z(SMA_{HT})$ sarcopenia cutoff value is simply − 2.0 according to the EWGSOP definition, although optimal z-score cutoff values should be investigated further. Individual studies using the same z-score equation could report whether the − 2.0 cutoff was optimal for a given cohort and clinical outcome.

The $z(SMA_{HT})$ score is quite simple to compute in practice (for simplicity, the coefficients in the following equation have been rounded). For each subject, *i* (*sex* = 1 if male, and 0 if female):

1. compute the $SMA_{HT}$ (cm^2^/m): $I_i = SMA_i/height_i$,
2. compute the predicted mean $SMA_{HT}$ from BMI and sex: $m_i = 50 + BMI_i + 13 \times sex_i + 0.6 \times BMI_i \times sex_i$,
3. compute the sex-specific standard deviation: $s_i = 8.8 + 2.6 \times sex_i$
4. compute the z-score: $z_i = (I_i - m_i)/s_i$.

For those who prefer to use the existing height-squared SMA normalization, computing the $z(SMA_{HT2})$ score would be similar:

1. compute the $SMA_{HT2}$ (cm^2^/m^2^): $I_i = SMA_i/(height_i^2)$,
2. compute the predicted mean $SMA_{HT2}$ from BMI and sex: $m_i = 30 + 0.7 \times BMI_i + 5.5 \times sex_i + 0.3 \times BMI_i \times sex_i$,
3. compute the sex-specific standard deviation: $s_i = 5.4 + 1.5 \times sex_i$,
4. compute the z-score: $z_i = (I_i - m_i)/s_i$.

Differences between sarcopenia cutoffs derived in different cohorts have been attributed to disease, ethnicity, or nationality while simultaneously reporting large differences in BMI and/or age^2,4,5,9,15,33^. Comparing sarcopenia cutoff values derived from studies using different age and BMI distributions is a flawed premise when both age and BMI are correlated with the metric used to define sarcopenia. Age and BMI distributions naturally vary between populations and study cohorts, and this is desirable. However, it is undesirable to require different sarcopenia cutoffs for every disease, ethnicity, or nationality, particularly if those differences can be explained by anthropometric factors. Indeed a visual examination of $SMA_{HT}$ versus BMI, split by self-reported race (Fig. S3), did not suggest that adjustment for race is necessary after adjusting for height and BMI. In other words, observed differences between cohorts should not be attributed to racial or ethnic differences without first controlling for age, sex, height, and BMI. To that end, we suggest that unbiased comparisons between cohorts of different age, sex, race, height, and BMI instead be made using z-scores calculated from our equations.

This study has important limitations. These equations do not apply to SMA measured at other vertebra levels or measured via other imaging modalities, they apply to CT-derived L3 SMA only. They also have not been tested for validity in children under age 18. While we hope that these equations accurately quantify the relationship between SMA, sex, height, and BMI in healthy, young adult (age 18–40) populations around the world, we cannot be sure, and this should be investigated further. The − 2.0 z-score cutoff has not been tested against clinical outcomes. We used non-contrast-enhanced CT scans; previous research has shown that IV contrast has a clinically insignificant effect on SMA^12,33,34^.

We propose that this z-score method, using these equations, be used as a consistent global framework for measuring and reporting height- and BMI-adjusted sarcopenic low L3 skeletal muscle area cutoffs across different cohorts.

## Methods

### Study cohort

We retrospectively studied persons who underwent CT scans at the University of Michigan as part of evaluation for kidney donation between 1999 and 2017. We have previously studied subsets of these kidney donor candidates as a healthy reference population^12,15^.

Patient age, sex, height (m), and weight (kg) were obtained from their medical record proximal to the date of evaluation for kidney donation, and the month and year of the evaluation appointment was recorded^35^. Candidates were included if they had a non-contrast-enhanced series CT scan performed as part of evaluation for kidney donation, with a complete fascia boundary visible in the display field of view, had age, sex, height, and weight recorded in their electronic medical record, and were medically, surgically, and psycho-socially approved for donation.

Body mass index (BMI) was computed and categorized into groups according the World Health Organization International Classification standard^36^. Self-reported race, unavailable for 31% of the cohort, was not specifically analyzed, however, it was used in a visual evaluation of the final z-score (Fig. S3).

CT imaging was extracted for 1849 total donor candidates between the ages of 18 and 68 scanned using the GE ‘Standard’ reconstruction algorithm at 120 kVp and 5mm slice thickness in a Discovery or LightSpeed scanner. Tube current was automatically modulated in proportion to body mass.

The study was split into two cohorts; the $n=1058$ ‘young adult’ candidates age 18–40 (‘Under-40’) and the $n=791$ candidates over age 40 (‘Over-40’).

### CT image processing

After being transferred into a spatial database, CT images were processed using Analytic Morphomics, a semi-automated image analysis method that has been previously described^12,37^. A combination of automated and user-guided algorithms written in matlab (The Mathworks Inc, Natick, MA) identified vertebral bodies. Next, the outer abdominal fascia and inner muscle wall were identified to create enclosed regions of interest, which were confirmed by multiple trained researchers.

SMA was measured as the area of pixels between − 29 and +150 Hounsfield Units (HU) in the region of interest on the axial slice nearest the inferior aspect of the third lumbar vertebral body (L3) as previously validated^12,33,38^.

### Statistical methods

Male and female demographics, CT parameters, and skeletal muscle measurements were summarized separately as mean and standard deviation (s.d.) for continuous variables and proportion for categorical variables. Means were compared using two-tailed t-tests assuming unequal variance and proportions were compared using the Chi-squared test.

Using the ‘Under-40’ cohort, sex-specific allometric regression models were fit to find the optimal integer coefficient for the relationship between weight versus height, and SMA versus height. The allometric model $SMA = \alpha height^{\beta }$ was transformed into the logarithmic form $log_e(SMA) = \alpha + \beta log_e(height) + \epsilon$ and linear regression was used to find the $\beta$ coefficient (optimal power of height)^39^. The resulting coefficient rounded to 1 as the nearest integer in both males and females, ergo two height-adjusted skeletal muscle indices were computed for comparison: the ‘standard’ SMI using a height power of two ($SMA_{HT2}=SMA/height^2$), and an alternative using a height power of one ($SMA_{HT}=SMA/height$), as suggested by allometric modeling.

For completeness, a BMI-adjusted measure ($SMA_{BMI}=SMA/BMI$, mathematically equivalent to $(SMA \times height^2)/weight$), and a weight-adjusted measure ($SMA_{WT}=SMA/weight$) were computed and analyzed, with results included in supplemental materials (Tables S1, S2, Fig. S1).

To describe the relationship between BMI and height-adjusted SMA in a young, healthy adult cohort, two multiple linear regression models were constructed using the ‘Under-40’ cohort; one for $SMA_{HT}$ and one for $SMA_{HT2}$. In each model, the height-adjusted index (I) was the response, while BMI, male sex, and their interaction were predictors, allowing for different intercept and slope by sex, e.g., ${\widehat{I}} = \beta _0 + \beta _1 \times BMI + \beta _2 \times sex + \beta _3 \times sex \times BMI$.

Finally, each index was converted into a z-score with mean zero and standard deviation one by subtracting the expected mean (predicted value from the regression equation) and dividing by the sex-specific ‘Under-40’ standard deviation (residual standard error from the regression equation), e.g., $z(I) = (I - {\widehat{I}})/SD(I)$.

The final regression equations were as follows (*sex* = 1 if male, and 0 if female):

$${\widehat{I}} = 30.02 + 0.67 \times BMI + 5.49 \times sex + 0.26 \times BMI \times sex$$, for $$I=SMA_{HT2}$$

$${\widehat{I}} = 49.98 + 1.07 \times BMI + 12.95 \times sex + 0.61 \times BMI \times sex$$, for $$I=SMA_{HT}$$

Sex and BMI explained 60% of the variation in $SMA_{HT2}$ and 73% of the variation in $SMA_{HT}$ (Adjusted $R^2$), with residual standard errors of 5.4/6.9 (Female/Male) and 8.8/11.4 (Female/Male) respectively, and all model coefficients statistically significant (*p* ≤ 0.01).

Bivariate scatter plots and Pearson correlation coefficients were used to assess the linear association between each skeletal muscle measure and age, BMI, height, and weight separately for males and females and each cohort.

An alpha level of 0.01 was used to determine statistical significance. All statistical tests were performed in R version 3.5.3^40^, using the package ‘ggplot2’^41^ for data visualization.

### Ethical approval and informed consent

This study was approved by the Institutional Review Board of the University of Michigan. All methods were performed in accordance with the relevant guidelines and regulations of the United States. Because existing CT scans were used retrospectively, the requirement for informed consent was waived by the Institutional Review Board of the University of Michigan.

## Supplementary information

Supplementary information.

## Data Availability

The datasets generated during and/or analyzed during the current study are available from the corresponding author upon reasonable request.
