# Supplementary material for: Optimal body size adjustment of L3 CT skeletal muscle area for sarcopenia assessment
Source: Sci Rep. 2021 Jan 11;11:279. doi: 10.1038/s41598-020-79471-z (PMC7801425; doi:10.1038/s41598-020-79471-z)
Supplement: Supplementary file 1 — Supplementary information. [file 41598_2020_79471_MOESM1_ESM.pdf]

Sex	Variable	Under-40 N	Under-40 mean	Under-40 s.d.	Over-40 N	Over-40 mean	Over-40 s.d.	p
F	SMA/BMI $(cm^2/(kg/m^2))$	610	4.90	0.86	512	4.50	0.75	$<$**.001**
	SMA/wt (cm^2/kg)	610	1.81	0.30	512	1.68	0.27	$<$**.001**
M	SMA/BMI $(cm^2/(kg/m^2))$	448	7.17	1.03	279	6.78	0.83	$<$**.001**
	SMA/wt (cm^2/kg)	448	2.24	0.32	279	2.14	0.25	$<$**.001**

Table S1. Sex- and cohort-specific alternative skeletal muscle measures adjusted for weight and BMI directly.

Cohort	Variable	Sex	N	Age	BMI	Height	Weight
Under-40	SMA/BMI	F	610	**-0.146 p<.001 (-0.222, -0.067)**	**-0.690 p<.001 (-0.729, -0.646)**	**0.361 p<.001 (0.290, 0.428)**	**-0.505 p<.001 (-0.562, -0.444)**
		M	448	**-0.219 p<.001 (-0.305, -0.129)**	**-0.643 p<.001 (-0.694, -0.585)**	**0.264 p<.001 (0.175, 0.348)**	**-0.465 p<.001 (-0.534, -0.389)**
	SMA/wt	F	610	**-0.121 p=0.003 (-0.199, -0.042)**	**-0.722 p<.001 (-0.758, -0.682)**	**-0.114 p=0.005 (-0.192, -0.035)**	**-0.718 p<.001 (-0.755, -0.677)**
		M	448	**-0.184 p<.001 (-0.272, -0.092)**	**-0.647 p<.001 (-0.698, -0.590)**	**-0.290 p<.001 (-0.373, -0.203)**	**-0.700 p<.001 (-0.744, -0.650)**
Over-40	SMA/BMI	F	512	**-0.271 p<.001 (-0.349, -0.189)**	**-0.673 p<.001 (-0.718, -0.623)**	**0.330 p<.001 (0.250, 0.405)**	**-0.494 p<.001 (-0.556, -0.425)**
		M	279	**-0.238 p<.001 (-0.345, -0.124)**	**-0.438 p<.001 (-0.528, -0.338)**	**0.369 p<.001 (0.263, 0.466)**	**-0.177 p=0.003 (-0.289, -0.061)**
	SMA/wt	F	512	**-0.253 p<.001 (-0.333, -0.170)**	**-0.659 p<.001 (-0.705, -0.607)**	**-0.131 p=0.003 (-0.215, -0.044)**	**-0.677 p<.001 (-0.721, -0.627)**
		M	279	**-0.229 p<.001 (-0.338, -0.115)**	**-0.458 p<.001 (-0.546, -0.360)**	**-0.283 p<.001 (-0.388, -0.172)**	**-0.537 p<.001 (-0.616, -0.448)**

Table S2. Sex-specific Pearson correlation, p-value, and 95% CI shown for alternative skeletal muscle measures adjusted for weight and BMI directly. Statistically significant correlations ($p < .01$) shown in bold.

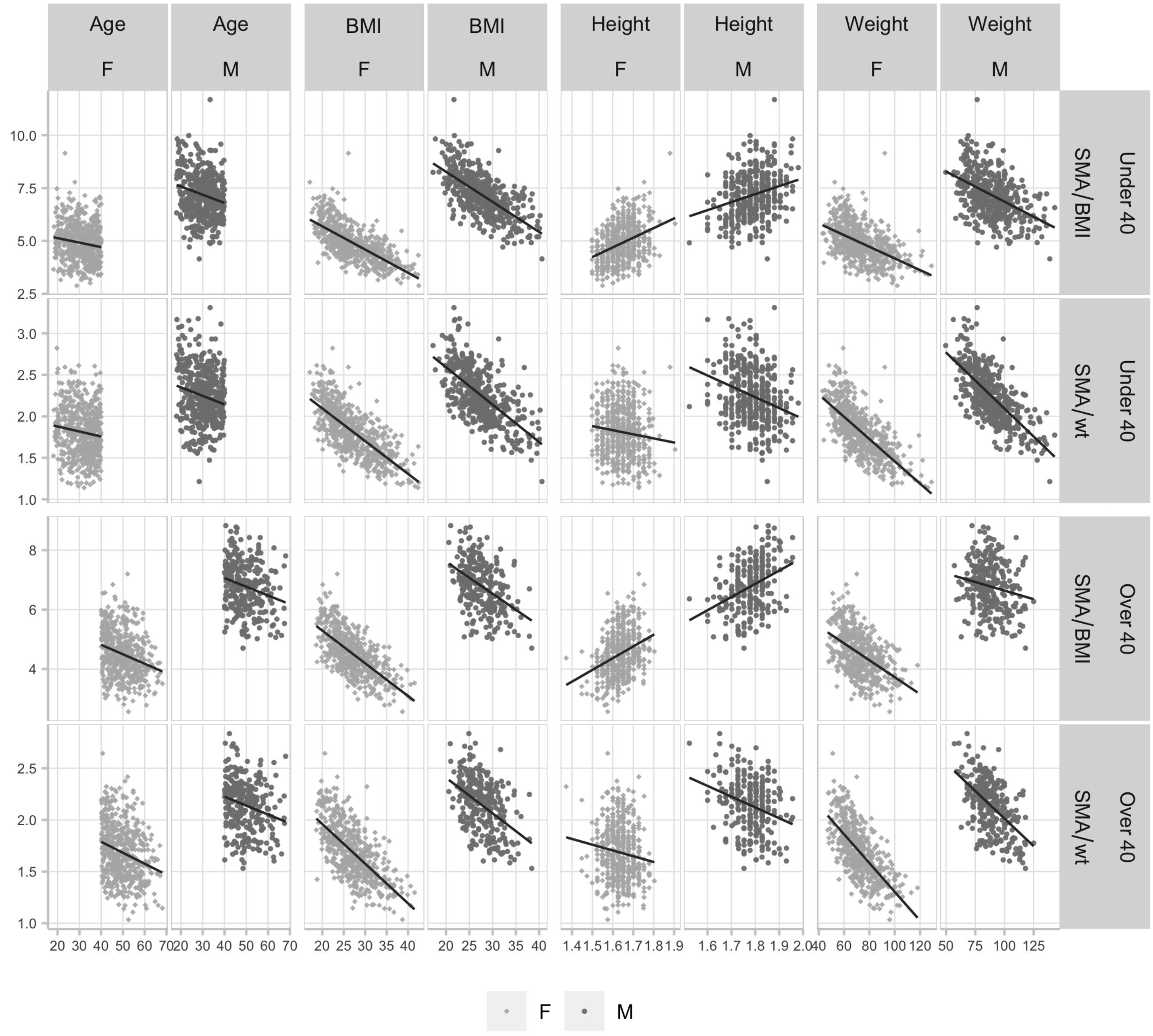

Figure S1. Scatter plots of alternative L3 skeletal muscle area measures versus age, BMI, height, and weight, split by sex. Best-fit linear regression line overlaid (black, solid) for both 'Under-40' and 'Over-40' cohorts.

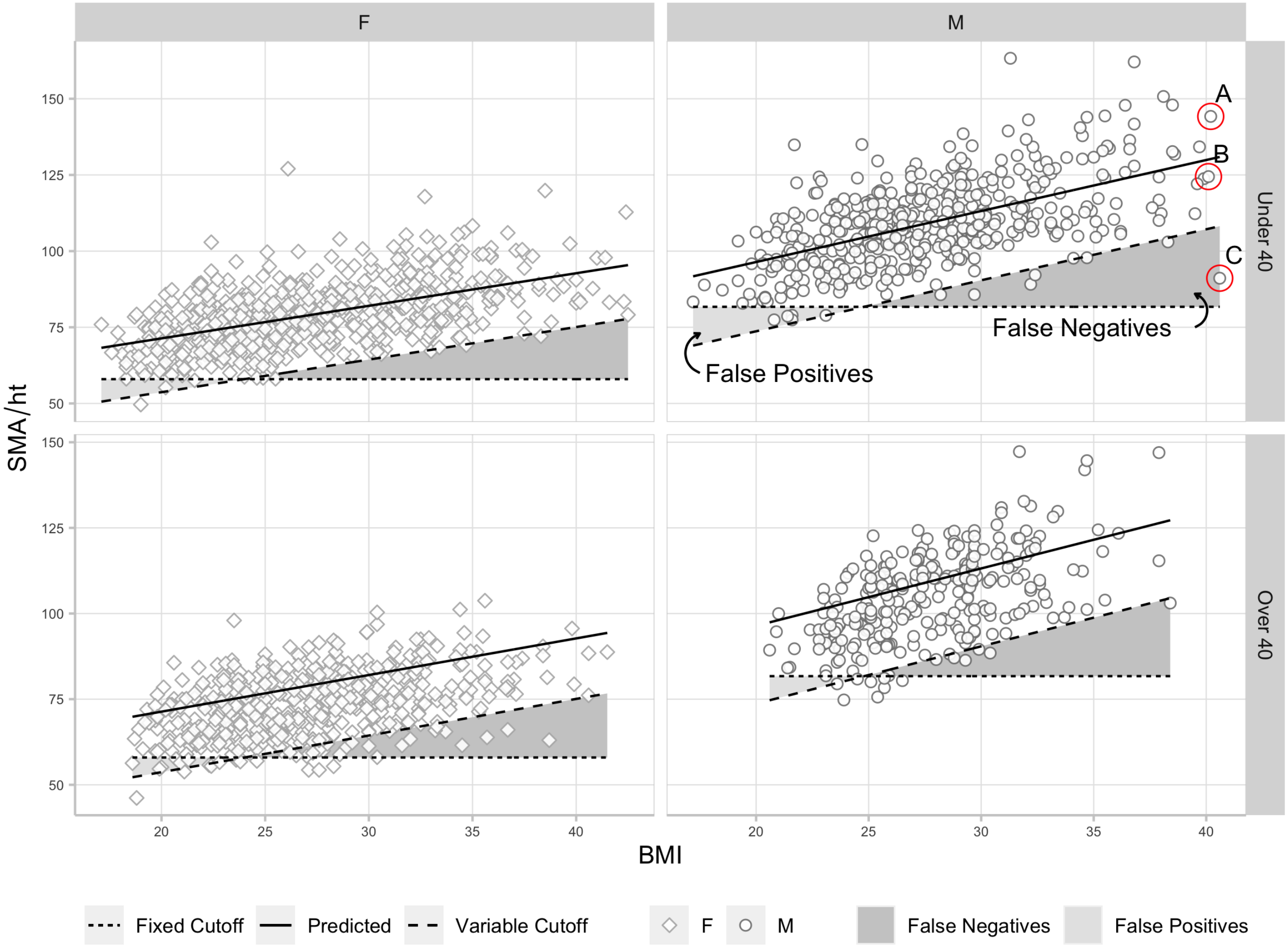

Figure S2. Alternative version of Figure 3, using SMA/ht fixed (mean - 2s.d.) cutoff values computed from the 'Under-40' cohort: 58.0 (Female), 81.7 (Male). Refer to Figure 3 for L3 axial CT images highlighting skeletal muscle area for three males of similar age (28-29) and BMI 40.1-40.6, but different $z(SMA_{HT})$ scores (A=1.23, B=-0.49, C=-3.50) are compared. The third individual (C) has a muscle area more than 3 s.d. below the mean value expected for a BMI of 40. He would be classified as not sarcopenic using the fixed cutoff, but would be sarcopenic using the variable (z-score) cutoff. Scatter plots show L3 skeletal muscle area divided by height (SMA_{HT}) versus BMI, split by sex and cohort. Lines overlaid for (1) the mean value predicted by our linear regression model (solid line), (2) sarcopenia 'fixed cutoff' values (dotted line), and (3) bmi-adjusted 'variable cutoff' values (dashed line) computed as two s.d. below the predicted mean. Regions of 'False Positives' and 'False Negatives' sarcopenia diagnosis are shown based on the difference between the 'fixed' and 'variable' cutoffs.

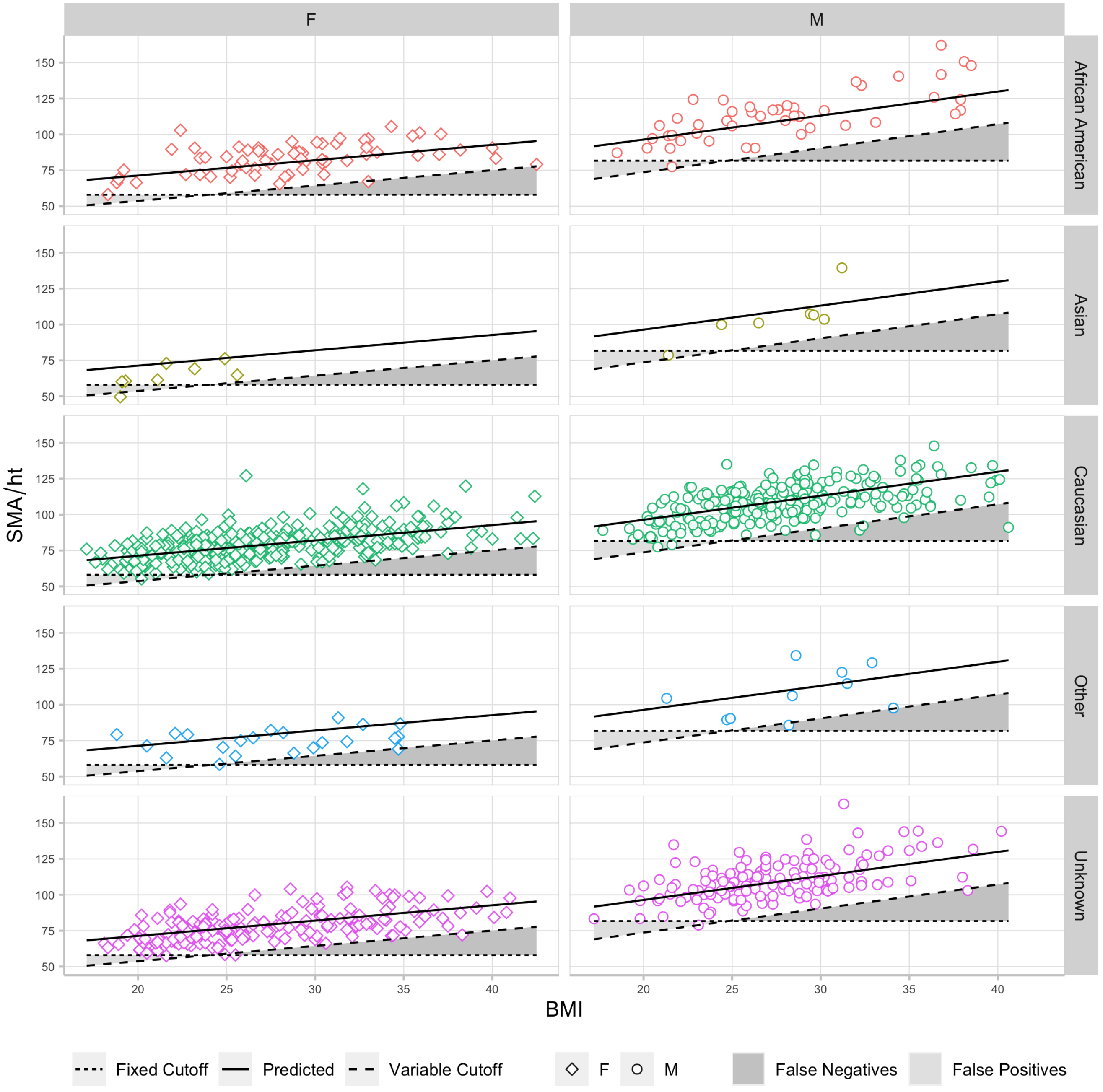

Figure S3. Scatter plots show L3 skeletal muscle area divided by height (SMA_{HT}) versus BMI, split by sex and race for the 'Under-40' cohort. Lines overlaid for (1) the mean value predicted by our linear regression model (solid line), (2) sarcopenia 'fixed cutoff' (mean - 2s.d.) values, 58.0 (Female) and 81.7 (Male), computed from the 'Under-40' cohort (dotted line), and (3) bmi-adjusted 'variable cutoff' values (dashed line) computed as two s.d. below the predicted mean. Regions of 'False Positives' and 'False Negatives' sarcopenia diagnosis are shown based on the difference between the 'fixed' and 'variable' cutoffs.
